# Application of solitaire double-stent thrombectomy in the treatment of accessory middle cerebral artery occlusion: a case report

**DOI:** 10.3389/fsurg.2025.1499918

**Published:** 2025-08-21

**Authors:** Qi Han, Guang-Hui Dong, En-Bo Zhu, Ming-Quan Lin, Guang-Lin Liu, Guang-lin Jin, Lin-Zhuo Qu, Hong-Jian Guan, Hui-ying Che

**Affiliations:** ^1^ Department of Neurology, Yanbian University Hospital, Yanji, Jilin, China; ^2^ Department of General Medicine, Yanbian University Hospital, Yanji, Jilin, China

**Keywords:** AMCA, solitaire, cerebral infarction, dissection, embolism, digital subtraction angiography, magnetic resonance angiography

## Abstract

The accessory middle cerebral artery (AMCA) refers to the cerebral vascular variation originating from the anterior cerebral artery, passing through the lateral fissure and accompanying the middle cerebral artery (MCA), and participating in the blood supply area of the MCA. Relevant literature reports that the incidence of this variant vessel is 0.3%–2.7% (autopsy) and 0.26%–4.0% (cerebral angiography), respectively. This article reports a case of acute MCA occlusion with AMCA mutation. The occluded main MCA was successfully opened using a Solitaire double-stent thrombectomy to avoid mistaking AMCA for the main MCA.

## Introduction

The accessory middle cerebral artery (AMCA) and duplicated MCA are fascinating variations in cerebrovascular anatomy. The AMCA refers to a cerebral vascular variation that originates from the anterior cerebral artery and passes through the lateral fissure to accompany the MCA, participating in the blood supply area of the MCA (1, 2). Understanding the presence and characteristics of the AMCA is crucial for accurate diagnosis and treatment planning in cases involving potential cerebrovascular disorders. The duplicated MCA is another interesting anomaly where there is an additional branch that mimics or partially duplicates the normal MCA. This duplication can have significant implications for understanding cerebrovascular physiology and pathology (3). It may pose challenges in diagnostic imaging and surgical interventions as it can be misidentified or lead to complex vascular patterns that require careful assessment. Studying these vascular variations not only enhances our knowledge of normal and abnormal cerebrovascular anatomy but also has practical implications for clinicians, radiologists, and researchers. It can help improve the accuracy of diagnoses, guide treatment strategies, and contribute to a better understanding of the underlying mechanisms of cerebrovascular diseases. This article mainly reports a case of a young person with occlusion of the MCA treated with double stent thrombectomy. Also, it provides a literature review on related literature.

## Case report

**Figure 1:**
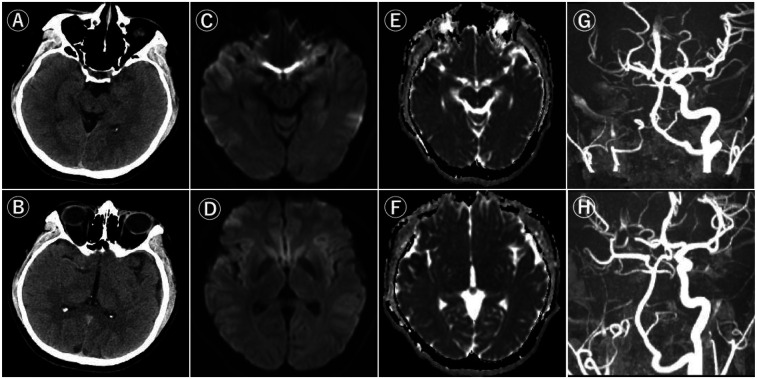
Computed tomography and magnetic resonance imaging.

A previously healthy 24-year-old man presented to our hospital with sudden onset of left-sided weakness, three hours after he was last known to be well. The time from the discovery of symptoms to our hospital was 30 min; The time from the patient's last normal time to arrival in our hospital was 50 min. At the time of physical examination, the patient had right common deviation, left hemiplegia, and left sensory impairment. The National Institutes of Health Stroke Scale (NIHSS) score was 7. Computed tomography (CT) showed that there was no obvious abnormal density area in the brain parenchyma at all levels, the ventricular system was normal, and the midline structure was in the middle. Diffusion-weighted imaging (DWI) showed patchy and patchy high signal intensity in the right frontotemporal lobe and basal ganglia. Magnetic resonance angiography (MRA) did not show the right internal carotid artery, and the right MCA was slightly slender with reduced signal (Figures 1A–H). We diagnosed acute cerebral embolism due to right ICA occlusion and recognized that DWI and flair with high signal intensity did not match. Therefore, we decided to perform an intravascular thrombectomy to relieve ICA occlusion. The patient's family members have informed the operation.

**Figure 2:**
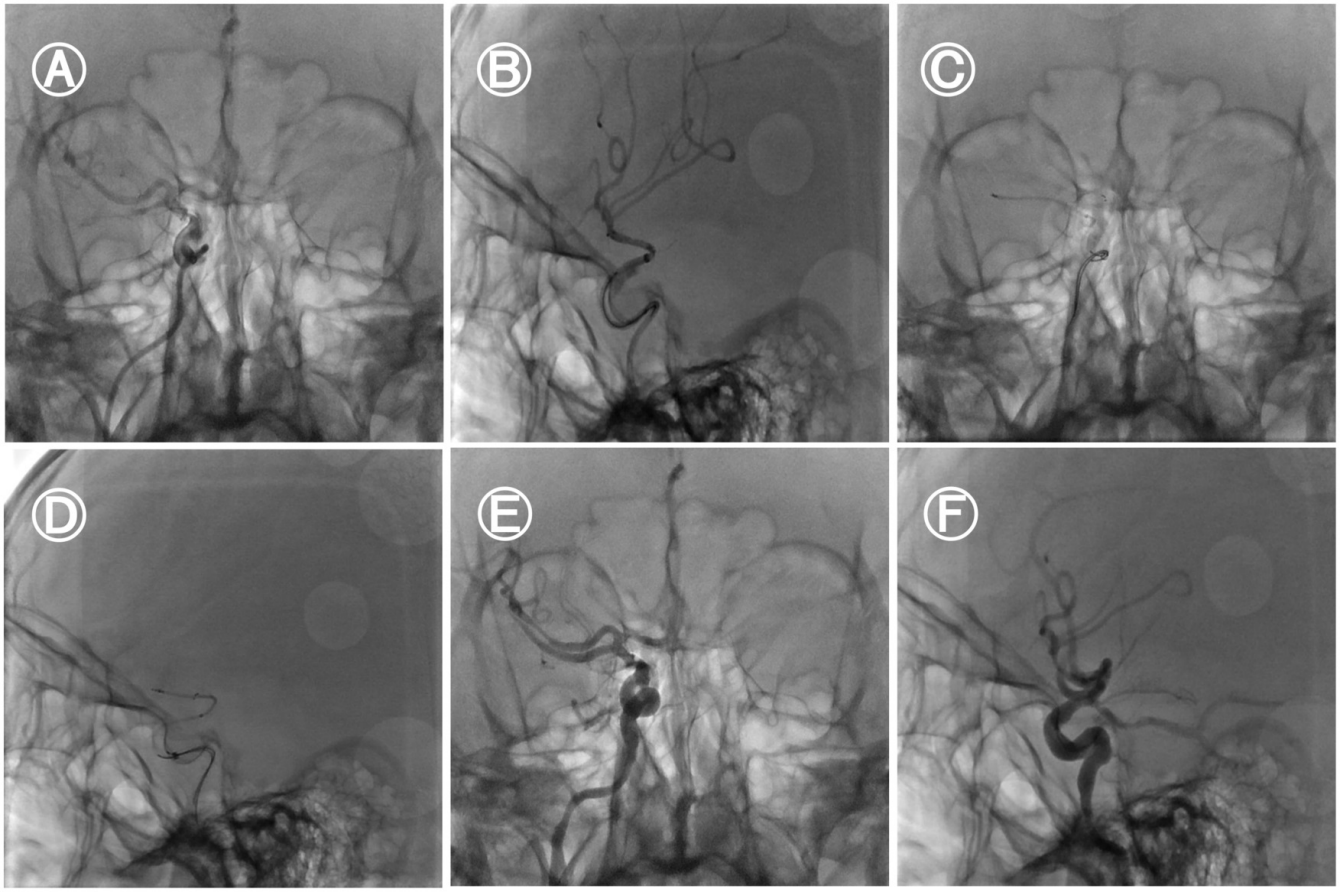
Digital subtraction angiography.

**Figure 3:**
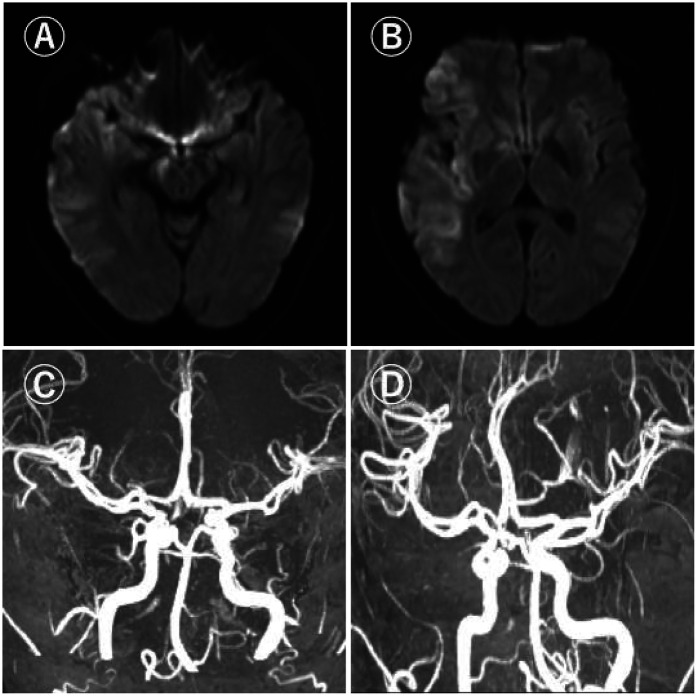
Postoperative magnetic resonance imaging.

Under general anaesthesia, the 8F sheath was retained after a successful puncture with the Seldinger technique. After cerebral angiography, it was found that the right internal carotid artery was occluded. The loach guide wire and multifunctional catheter were used to guide the 8F guide catheter to the right internal carotid artery. The loach guide wire and multifunctional catheter were withdrawn. The micro-guide wire reached the M2 segment of the right MCA through the occlusion of the right MCA. The micro-guide wire was guided to send the intermediate catheter to the right internal carotid artery. The micro-guide wire was guided to send the micro-catheter to the V1 segment of the right MCA, and the micro-guide wire was withdrawn. Under fluoroscopy, the SFR 4.0 mm × 20 mm stent was carefully sent to the distal end of the M1 segment, and the stent was released. The branches of the right MCA were well developed in the reexamination angiography. On the basis of the stent anchoring, the intermediate catheter was followed up to the right MCA M1 segment, and then the thrombus was pulled while the suction was performed. No thrombus was found in the thrombectomy stent and the suction catheter. The micro-guidewire carefully reached the A2 segment of the right anterior cerebral artery through the occlusion of the right anterior cerebral artery. The micro-guidewire guided the micro-catheter to the distal end of the A2 segment of the right anterior cerebral artery, and the micro-guidewire was withdrawn.Under fluoroscopy, the SFR 4.0 mm × 20 mm stent is meticulously delivered to the distal end of the A2 segment of the right anterior cerebral artery and released. Subsequently, the microcatheter is withdrawn from the body. Then, guided by the microwire, the microcatheter traverses through the first stent and enters the MCA. Thereafter, via the microcatheter, the SFR 6.0 mm × 30 mm stent is delivered to the right MCA and released. On the basis of stent anchoring, the intermediate catheter is advanced to the communicating segment of the right internal carotid artery. After 5 min, the thrombus was pulled at the suction side, and the branches of the right internal carotid artery were well developed (Figures 2A–F). The thrombectomy achieved complete recanalization, which was defined as thrombolysis with a cerebral infraction score of 3 (TICI 3) (door to puncture, 85 min; puncture to recanalization, 108 min). Post-thrombectomy carotid angiogram identified AMCA, which arose from the proximal A1 segment of the left ACA. No bleeding was found in the head CT after the operation. After the thrombectomy, the patient's left co-directional deviation left hemiplegia and partial sensory disturbance were completely improved. Following the surgery, a head MRI reexamination was carried out, revealing that AMCA and the main vessels of the MCA had been recanalized (Figures 3A–D). The NIHSS score was 6 the day after the thrombectomy and 0 at discharge after 10 days of hospitalization. This case report was approved by our hospital's ethics committee with the informed consent of the patient's family members.

## Discussion

Variations in the MCA pose one of the current challenges in the treatment of stroke during the ultra-acute stage. Mechanical thrombectomy has emerged as a standard and effective treatment strategy for acute ischemic stroke caused by large vessel occlusion, but it is not applicable to all stroke subtypes (4, 5). For patients with cerebral infarction resulting from vascular variations, relevant research reports suggest that endovascular treatment might lead to adverse outcomes. Hence, prior to endovascular interventional therapy, a detailed hemodynamic assessment is essential. Particularly when there is a mismatch between clinical symptoms and imaging findings as indicated by NIHSS scores or when complete occlusion of the internal carotid artery (ICA) does not correlate with the patient's clinical symptoms, clinicians should contemplate MCA vascular variations in such cases (6).

Among patients with acute ischemic stroke, especially those with large artery occlusion, embolism represents one of the leading etiologies. Typically, the common sites of embolism are locations where the arterial course alters, such as the top of the ICA, the tip of the basilar artery, the bifurcation of the MCA, and atherosclerotic lesions. A clinical investigation revealed that among reported cases of accessory MCA occlusion (7), cardiogenic embolism accounts for the highest proportion, followed by atherosclerosis, cerebral artery dissection, and embolism of unknown origin. The accessory MCA generally has a smaller diameter than the M1 segment of the normal MCA, which is closely related to the vascular anatomical structure. Consequently, the likelihood of embolism at this location is relatively high. At present, many scholars have discovered in studies on the anatomical structure of the accessory MCA that the incidence of AMCA is 0.3%–4.0%, and that of DMCA is 0.2%–2.9% (8). Therefore, it is rather challenging to accurately determine whether it is AMCA- or DMCA-related acute ischemic stroke and conduct vascular recanalization treatment as early as possible. If we cannot identify the vascular anatomy in cases of ICA occlusion involving DMCA or AMCA, then after revascularization surgery, even though DMCA or AMCA is occluded, we may misjudge the recanalization of ICA and MCA.

At present, there are reports on the concept of using ADAPT treatment in cases of ICA occlusion combined with MCA and DMCA occlusion. The case in this study employed the Solitaire double stent thrombectomy technique, which is similar to that used by Koge et al. (3). This is because when the vascular anatomical structure is unclear, it is challenging for patients with acute ischemic stroke and large vessel occlusion accompanied by bifurcation to promote sufficient revascularization during standard mechanical thrombectomy. Consequently, mechanical thrombectomy using a double stent retriever in these individuals may be an effective salvage treatment (9). Large vessel occlusions involving bifurcations typically have persistent clot attachments, leading to a decreased probability of successful recanalization. In such cases, the application of a single stent retriever or other salvage therapies such as intra-arterial thrombolysis, thrombus aspiration, balloon angioplasty, or intracranial stent placement may not achieve recanalization of all branches. The double stent thrombectomy technique has a relatively high recanalization rate. Simultaneously, since the double stent thrombectomy technique has a larger surface area of the device, it can enhance the adhesion at the distal end of the thrombus and increase the likelihood of the thrombus being pulled out (10, 11). For instance, in this surgical procedure, the first step involves initially delivering the first stent to the right anterior cerebral artery and deploying it. Subsequently, the microcatheter is withdrawn from the body. Thereafter, guided once again by the microwire, the microcatheter traverses through the first stent and enters the right MCA. Subsequently, via the microcatheter, the second stent is released.Certainly, there is another approach. A 3-meter microwire is reserved in the MCA. Another microwire is used to guide the microcatheter to reach the anterior cerebral artery. A stent is released and the microcatheter is removed. The removed microcatheter reaches the MCA along the reserved 3-meter guidewire. After removing the microwire, the second stent is released into the MCA. In other words, the two stents are released in parallel. Naturally, the double stent thrombectomy technique has some potential drawbacks, such as vascular injury, dissection, endothelial wall damage, and arteriole avulsion. The potential risk is higher than that of simple stent technology (12). Moreover, this research result is also in line with the conclusion of Jiang et al. (13). The microcatheter can act as a guiding instrument to facilitate the accurate placement of the stent retriever and prevent it from moving and entangling randomly within the blood vessel. Through the precise guidance of the microcatheter, the position and direction of the stent retriever can be better controlled. Simultaneously, following up with the intermediate catheter to the end of the stent can exert a better stabilizing effect, thereby enhancing the success rate and safety of the surgery. During the operation, closely observe the angiographic images and adjust the positions of the microcatheter and stent retriever in a timely manner to ensure the smooth progress of the surgery.

Taichiro et al. (14) further demonstrated the feasibility of this approach in their study on double stent thrombectomy. A recent meta-analysis further confirmed that double stent thrombectomy achieved an mTICI≥2b effect in 92.6% of cases, and the first-pass effect reached 76.6% (15). Currently, there are five common anatomical variation types of AMCA or DMCA reported, including accessory MCA, duplicated MCA, double-origin MCA, fenestrated MCA, and branched MCA (16). The patient reported in this case is of accessory MCA. After cerebral angiography, a bifurcation was found at the origin of the anterior cerebral artery, and there was obvious local stenosis in the M1 segment of the right MCA. After considering the presence of an accessory MCA, double stents were used for thrombectomy. The forward blood flow was significantly improved after the operation (TICI grade 3). Thus, during the thrombectomy process, we should do our best to identify the anatomical variations of AMCA or DMCA. In a study of 20 existing reports (17), it was found that the average age of patients with AMCA or DMCA cerebral infarction is 55.8 ± 21.2 years old (mean standard deviation; age range 21–84 years old), and the clinical NIHSS score is relatively mild (NIHSS < 8), accounting for 66.6%, with a good prognosis. After the patient, in this case, was treated with double stent thrombectomy, the NIHSS score at discharge was 0, and the mRS score was 1, indicating a favourable prognosis. This result is also consistent with the reported results mentioned above. In the etiological analysis of this patient, it was found in the follow-up and reexamination after 3 months that the right heart contrast echocardiography result of this patient showed a positive foaming test, suggesting the presence of a right-to-left shunt. Patent foramen ovale was considered, and the possibility of pulmonary arteriovenous fistula could not be excluded. The patient went to Beijing Tiantan Hospital, Affiliated with Capital Medical University, to undergo patent foramen ovale closure in the same month. Therefore, in the case of young patients with large vessel occlusion, during the process of excluding risk factors, it is necessary to highly suspect the possibility of patent foramen ovale and pulmonary arteriovenous fistula.

In summary, we report a case of a patient with acutely occluded AMCA who underwent arterial thrombectomy. We employed the Solitaire double stent thrombectomy technique. This is because double stent thrombectomy can effectively reduce the frequency of thrombectomies, increase the vascular recanalization rate, and not increase the risk of symptomatic haemorrhage while improving the favourable prognosis (11). Nevertheless, its safety remains open to discussion. Additionally, double stent thrombectomy may result in a significant increase in the hospitalization cost for acute stroke treatment and a large number of sample studies are required to determine its cost-effectiveness further.

## Data Availability

The datasets presented in this article are not readily available because of ethical and privacy restrictions. Requests to access the datasets should be directed to the corresponding authors.
